# Validation of the web-based dietary assessment tool (RiksmatenFlex) against doubly labelled water and 24-h dietary recalls in Swedish pre-school children

**DOI:** 10.1186/s12937-026-01315-9

**Published:** 2026-03-17

**Authors:** Anna Karin Lindroos, Jessica Petrelius Sipinen, Jennifer Rood, Marie Löf, Hanna Augustin, Michael Hoppe, Lotta Moraeus

**Affiliations:** 1 Department of Food Studies, Nutrition and Dietetics, Uppsala university, Box 560, Uppsala, SE-751 22 Sweden; 2 Department of Risk Benefit Assessment, Swedish Food Agency, Uppsala, Sweden; 3 Pennington Biomedical Research Center, Baton Rouge, LA USA; 4 Department of Medicine Huddinge, Karolinska Institute, Stockholm, Sweden; 5 Department of Internal Medicine and Clinical Nutrition, the Sahlgrenska Academy, University of Gothenburg, Gothenburg, Sweden

**Keywords:** Diet assessment, Energy intake, Energy expenditure, Food intake, Young children

## Abstract

**Background:**

RiksmatenFlex, a Swedish’ web-based dietary assessment method, has previously been validated in adolescents and pregnant women. The aim of this study was to evaluate RiksmatenFlex in preschool children by comparing energy intake with total energy expenditure (TEE) measured by the doubly labelled water (DLW) method, by comparing reported intakes of energy, foods, macro- and micronutrients with 24-h dietary recall interviews, and by assessing the parents’ perceptions of reporting their child’s dietary intake.

**Methods:**

Participants were 94 healthy children, 37 ± 15 months old, 51% girls, recruited through advertisements (preschools/social media). Energy, selected nutrient and food group intakes were assessed using RiksmatenFlex as a food diary and 24-h dietary recall interviews. TEE (DLW) was measured in a subsample of 37 children.

**Results:**

Average energy intake was overestimated by RiksmatenFlex compared to TEE (796 kJ; 21%; *P* < 0.001) in the subsample. The Bland-Altman plot showed wide limits of agreement (± 1797 kJ), and bias towards overreporting at higher intake levels (*r* = 0.34; *P* = 0.038). Energy intake and absolute intakes of protein, fat, carbohydrates, calcium (also energy standardised) and fruit-vegetables (combined) were significantly higher with RiksmatenFlex than with the 24-h dietary recalls. Other nutrient and food group intakes were fairly similar with the two methods. Spearman rank correlations ranged from 0.73 (wholegrains) to 0.98 (baby foods). Agreement between the two methods was substantial (K_W_>0.61) for most dietary variables, but not wholegrains, and proportion of energy from macronutrients (K_W_ 0.50–0.60). Parents found reporting food intake in RiksmatenFlex easy, with most preferring on-line reporting over interviews.

**Conclusion:**

The current study showed reasonable validity in young children and the method was well accepted by parents. These finding support its application in large-scale studies of young children, though potential overreporting of energy intake warrants attention.

**Supplementary Information:**

The online version contains supplementary material available at 10.1186/s12937-026-01315-9.

## Introduction

Information on dietary intake is crucial in nutrition research. This is particularly important in children, where detailed information on food consumption is needed to determine their nutritional requirements for sustaining growth and maintaining normal body function and health [1]. Knowledge on children’s food intake is also needed when developing and evaluating nutrition recommendations and food based dietary guidelines, and for use in dietary exposure assessments [2]. Furthermore, dietary habits formed early in life may have considerable impact on long-term health status [3] calling for early monitoring.

Measuring dietary intake correctly is, however, challenging. Accuracy and precision of most dietary assessment methods have been reported to be low in both adults [4–6] and children [1, 7, 8]. Assessing the diets of young children is further complicated by the dependency on the children’s caregivers and their ability to report their child’s food intake correctly. Parents may reliably report what their children eat in the home setting, but other caregivers involved in the reporting process are likely to approach the task with varying levels of motivation and interest [7].

The doubly labelled water (DLW) method [9], that measures total energy expenditure (TEE) over 10–14 days, is commonly used to validate reported energy intake of dietary assessment tools. Validation studies comparing energy intake with DLW energy expenditure show mixed results in children [1, 7, 8]. To exemplify, energy intake from food diaries that are filled in prospectively is generally underestimated in children [1, 7, 8], but the underreporting is also influenced by type and number of recorded days, age range, and health status [1]. In contrast, energy intake from dietary recalls, food frequency questionnaires and diet histories may, on the other hand, provide accurate estimates at the group level [1, 7, 8]. However, limits of agreement for estimated intakes between studies are still wide and there is substantial heterogeneity between studies [1, 7, 8].

The European Food Standards Agency (EFSA) recommends the dietary record method be carried out on two non-consecutive days (on paper or digital) for collecting food consumption data in children younger than 10 years old [10, 11]. The main reason for recommending food diaries is that different caregivers (at home, nurseries, schools) may have to be involved when recording children’s food intake. Administering food records on paper requires substantial staff resources. New technology and the wide internet use have made it possible to develop user-friendly dietary assessment methods where the recording is transferred from the investigators to the participants. This simplifies the data collection for researchers and makes it possible to scale up data collections and obtain detailed dietary information from large population groups.

The web-based method RiksmatenFlex was developed for the Swedish national dietary survey of adolescents in 2015 by the Swedish Food Agency (SFA) [12]. The dietary part of the method is flexible and can either be used as a prospective food diary or retrospectively as a 24-h dietary recall [12]. Previous validation studies have shown reasonable validity of the web-tool used as a 24-h dietary recall in adolescents [12] and pregnant women [13]. The web-based method has since then been updated to fit the registration of young children’s diets by adapting the food list and portion sizes of young children’s food intake.

### Objectives

The aims of this study were to validate reported energy intake by the RiksmatenFlex food diary against total energy expenditure measured by the DLW method in young children (aged 1–5 years); estimate the relative validity of RiksmatenFlex by comparing reported intakes of energy, foods, macro- and micronutrients with intakes obtained by 24-h dietary recall interviews; and finally, to describe the parents’ perceptions of reporting their child’s dietary intake by the two methods.

## Material & methods

### Study population

A convenience sample of 94 children were recruited through advertisements at preschools and on social media (i.e., Instagram and Facebook) from November 2022 till June 2023. Advertisements were restricted to areas close to the Swedish cities Uppsala and Gothenburg where the research centres were located. Inclusion criteria were child aged between 1 and 5 years and exclusion criteria were parents not understanding Swedish. The child’s mother or father served as the primary contact (contact parent). Written informed consent was collected from both parents before starting the study. Participants were compensated with a €20 gift card for the dietary part of the study and an additional €20 for the DLW part. Ethical approval was granted from the Swedish Ethical Review Authority (2020–05293).

### Study design

**Fig. 1:**
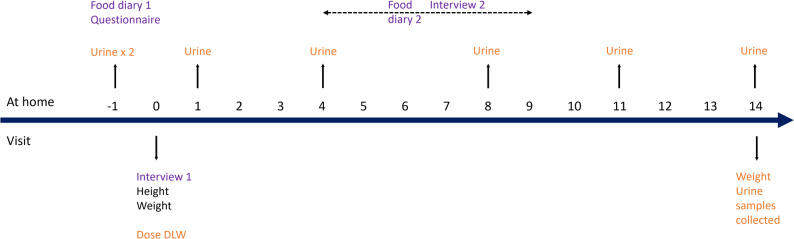
Overall study design. All participants (*n* = 94) conducted two food diary days (RiksmatenFlex) followed by two 24-h dietary recalls (Interview 1 and 2). The participants in the subgroup (*n* = 37) were administered an oral dose of doubly labelled water and collected urine samples over a period of 14 days

Food intake was reported in the web-based food diary RiksmatenFlex on two non-consecutive days and also reported in two 24-h dietary recall interviews covering the same days. Reported energy intake by both methods were validated against DLW in a subgroup of 37 participants. The relative validity of RiksmatenFlex was assessed by comparing the reported intake of energy, macro- and micronutrients and food groups with the reported intake by the 24-h dietary recalls. The overall design of the study is illustrated in Fig. 1. The contact parents of all children were scheduled for the first 24-h dietary recall interview, either face-to-face (DLW subgroup) or by telephone/video call (diet only). The second diet day occurred 5–10 days later and all interviews were conducted over the phone or via video call. The contact parent was instructed to record food intake in the RiksmatenFlex application the day before the dietary recall interview. Weight and height were reported by the parent for the diet only part of the study. The children and contact parents in the DLW subgroup (*n* = 37) were scheduled to visit the research centre for the first 24-h dietary recall interview, weight and height measurements and administration of the DLW dose. Participants in the DLW subgroup collected urine throughout the study and returned for a second visit to weigh the child and to provide the urine samples.

### RiksmatenFlex

The RiksmatenFlex method consists of a web page with a questionnaire part and a dietary assessment part. Dietary intake can either be reported retrospectively (24-h dietary recall) or prospectively (food diary) [12]. The RiksmatenFlexDiet used as a 2-day food diary (from now on called RiksmatenFlex) was updated for assessing dietary intake in the national survey Riksmaten Young Children 2021-24. Foods commonly consumed by toddlers and young children were added to the RiksmatenFlex food list using statistics of sales in this food segment as well as gathering menus from preschools. Portion sizes were also adjusted to young children by adding halved portions to the smallest eligible portions and by adding teaspoons to the household measures. Some new photos were also taken of foods commonly consumed by Swedish children (e.g., Falukorv a Swedish sausage). See Supplementary Material 1 for an overview of the method. In line with the EFSA recommendation for children below the age of ten, the method was administered as a food diary [10]. The parent registered the child’s intake of foods and drinks during two non-consecutive days. The second day was randomly assigned to occur 5–10 days after the first day. In the food diary, the parent added foods or dishes and chose portion sizes from photos or household measurements. A list of easily forgotten foods such as condiments and vitamin D supplements was presented at the end of the registration. There were also automatic reminders when a main meal (breakfast, lunch, dinner/evening meal) had less than four items and/or no drink recorded. It was expected that many children would attend preschool and have several of their meals there. A paper diary was therefore distributed to the parent with instructions to ask the preschool teachers to record everything eaten at the preschool and then record this intake afterwards in the on-line diary. A detailed description of the adaptation of the method for young children can be found elsewhere [2]. The food list of 1218 foods and composite dishes was connected to the SFA’s Food Composition Database (version Riksmaten Young Children) which enabled automatic assessment of energy and nutrient intake.

### 24-h dietary recalls

The two 24-h dietary recalls followed a standardized multiple pass protocol and was performed by two trained interviewers [14]. The parent was first asked to give an overview of times and intake of foods and drinks that were consumed by the child during the previous 24 h. They were then asked to give a more detailed account of each food item (i.e., amounts, portion sizes, ingredients of composite dishes, cooking methods etc.). Lastly the interviewer summarized the list of foods and revised if needed. To assist with portion sizes the participants could use a portion guide published by the SFA with photos of varying portion sizes presented for different foods [15]. Household measures and standard portion sizes of for example fruits (small, medium, large) were also used to aid the portion size estimations. The interviewer entered the food items into the software programme Dietist Net Pro (Kost och Näringsdata AB, Bromma, Sweden) and nutrient and food group intake was calculated using a the SFA Food composition database, version 2022-05-24 plus 547 additional foods specifically developed for dietary surveys by the SFA, yielding in total 2638 foods.

### Intake of nutrients and food groups

Energy, protein, fat, carbohydrates, added sugars, dietary fibre, wholegrains, vitamin C and D, iron and calcium were selected for the nutrient comparisons between the two dietary methods as important indicators of diet quality. Nutrient intakes are described in grams and standardised for energy intake (percent of total energy, E%, or grams per 1000 kJ). In addition, the food groups: fruits, vegetables, fruit juice, meat, processed meat, milk & yoghurts, cheese, bread, beverages, candy & chocolates and sweet pastries, and baby foods were selected for the comparisons in grams as they represent key foods eaten by young children. The food groups are presented in more detail in Supplementary Material 2 (Table S1).

### Parents’ perception of the dietary assessment methods

At the second 24-h dietary recall interview, the parents were also queried about their perception of the two different dietary assessment methods using the question “How easy was it to report your child’s food intake on a scale from one (very easy) to ten (very difficult)?” They were also asked what they found easy/difficult with the respective method and which method they preferred. For RiksmatenFlex they were also asked whether there were any foods that were difficult to find and how much time they spent on reporting foods in the food diary. The interviewer also recorded the time spent on the recall interview (not including time for entering foods into the software program Dietist Net Pro).

### Anthropometric measurements and background information

For the participants in the DLW subgroup, height and weight were measured according to standardized protocols at the research clinic. Weight was measured in light clothing to the nearest 0.1 kg using SECA 862 or 899 digital weighing scales. Height was measured without shoes to the nearest 0.1 cm using a SECA 213 portable stadiometer. If the children participated in the diet only part of the study, the parents were asked to provide their children’s weight and height from the latest health care visits (*n* = 21) or, if there was no recent visit, measure weight at home and estimate the height (*n* = 36).

For children 24 months and older BMI was calculated (kg/m^2^), and weight status was determined using the BMI-cut offs from the International Obesity Task Force [16]. Information on the child’s sex and birth date was collected at recruitment and the parents’ educational attainment was collected through the on-line questionnaire in the RiksmatenFlex application.

### Doubly labelled water

Total energy expenditure was measured over a 14-day period with the DLW method. This was done in parallel with the dietary assessments (Fig. 1). Two baseline urine samples were collected the day before the first visit when the child was given an accurately weighed dose of stable isotopes to drink. The DLW dose consisted of 0.20 g ^2^H_2_O (99.96%) and 0.35 g ^18^O (10%) per kg body weight. Urine samples were then collected at home on days 1, 4, 8, 11 and 14. Each sample was marked with a date and time for the sampling. The parents received verbal and written instructions on how to collect the urine. If the child wore diapers, parents were provided with detailed instructions on how to collect the urine by placing cotton balls in the diaper and then extracting the absorbed urine using a syringe (material provided by the study team). The samples were refrigerated until brought to the researchers. After 14 days the participants visited the research centre again and returned the urine samples. The child was also weighed again. The children were weight stable during the urine collection period. Collected urine samples were stored (-18°C) at the SFA in Uppsala until analysis. Samples were shipped to, and analysed at Pennington Biomedical Research Center, Baton Rouge, Louisiana, the United States. Each sample was analyzed for ^18^O and ^2^H abundance by isotope ratio mass spectrometry using automated devices for deuterium and ^18^O (GasBench/Delta Q, Thermo). The isotopic enrichments of the post-dose urines compared with the pre-dose samples were used to calculate elimination rates (k_d_ and k_o_) using linear regression. CO_2_ production was calculated using the equations of Speakman et al. [17]. Total energy expenditure was calculated by multiplying rCO_2_ by the energy equivalent of CO_2_ based on the estimated food quotient of the diet (0.86) at each time point.

### Statistical analysis and power considerations

The Stata statistical software release 18.5 (Stata Corp LP, College Station, Texas, USA) was used for the statistical analyses. In the DLW subsample, Bland-Altman plots, including limits of agreement (LOA) between methods, were used to assess precision and bias between reported energy intake and TEE calculated from the DLW method. Reported energy intake from the 24-h dietary recalls was also evaluated as this method was used as a reference in the relative validation. Pearson correlations were used to assess the correlation between reported energy intake and TEE, and whether there was a systematic bias between method means and method differences. Regression analysis was also used to determine the slope and intercept of the regression line. The calculated TEE from the DLW method reflects TEE over a two-week period, whereas energy intake was only reported on two non-consecutive days. Thus, the energy intake by the two dietary assessment methods was transformed from current intake to long-term intake using the statistical method Multiple Source Method, MSM [18, 19]. In the comparisons between the two dietary assessment methods the means of two days was used and the results are presented as median and interquartile range (IQR). Wilcoxon Signed Ranks Tests and Spearman Rank Correlations were used to test the differences and correlations between the two methods as most of the variables were not normally distributed. Agreement of reported intakes of energy, nutrient and food group intake by the two dietary assessment methods was also calculated by tertiles. Linear weighted kappa (κ_w_) was used to evaluate the agreement between the two methods. Power was difficult to assess for the DLW sub study as agreement rather than an absolute difference was the main outcome. Earlier DLW studies in young children have shown that it is possible to assess agreement and bias at the group and individual level using sample sizes of between 30 and 40 children [20, 21]. For the comparisons between the dietary assessment methods the aim was to recruit at least 92 children to be able to stratify the analyses by age group. This was based on the assumption that 46 children would be needed to detect an energy intake difference of 600 kJ, with a statistical power of 0.80.

## Results

### Study participants

Characteristics of all study participants and the DLW subsample are displayed by sex in Table 1. Mean age was 37 months, ranging from 12 to 68 months and mean weight was 15 kg (range 9–23 kg). The majority of contact parents were mothers (89 of the 94 contact parents), and 86% of the participants’ mothers and 63% of their fathers had more than 12 years of education. Age, weight, height, overweight/obesity, parents’ educational attainment, and reported energy intake by the two methods did not differ between the DLW subgroup and the diet only group, with no statistically significant difference observed.

**Table 1:** Characteristics and reported energy intake^a^ by the two dietary assessment methods of all children and the children in the doubly labelled water subsample

	All children, *n* = 94		DLW subsample, *n* = 37	
	Mean	SD	Mean	SD
Age, months	37	15	37	15
Weight, kg	15.2	3.3	15.7	3.2
Height, m	0.95	0.11	0.96	0.11
RiksmatenFlex, kJ	5134	1456	5370	1526
24-h dietary recalls, kJ	4915	1332	5086	1349
	**n**	**%**	**n**	**%**
Sex, girls	48	51	23	62
Overweight/obesity^b, c^	9	13	5	19
Higher education^d^				
Mothers	81	86	34	92
Fathers^e^	59	63	25	68

^a^Mean of two days, unadjusted values

^b^Based on the International Obesity Taskforce references [16]

^c^Children ≥ 24 months, *n* = 68 in total sample and *n* = 27 in subsample

^d^ >12 years of education

^e^Information from one father is missing

### Validity of reported energy intake in the DLW subsample

**Fig. 2:**
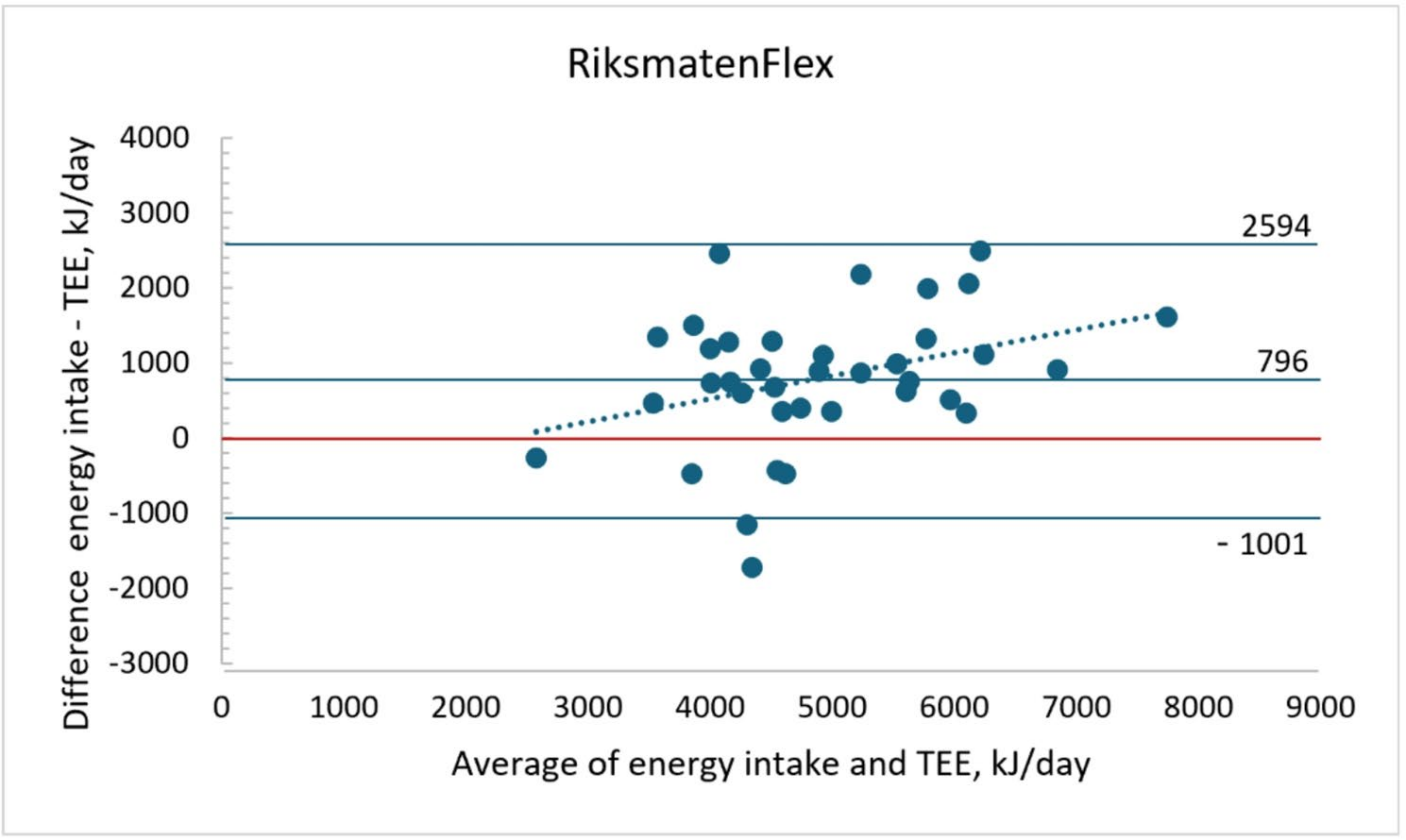
Bland-Altman plots of reported energy intake with RiksmatenFlex (mean of two days food diary) and total energy expenditure by doubly labelled water (TEE) (*n* = 37) (y = 0.31x – 716; *r* = 0.34, *P* = 0.038)

Mean (SD) total energy expenditure (TEE) by the DLW method was 4507 (980) kJ and reported energy intake with RiksmatenFlex was 5368 (1282) kJ. Energy intake was thus over reported with 796 kJ (18%, *P* < 0.001). The correlation between reported energy intake and TEE was 0.70. The agreement between reported energy intake and TEE is displayed in a Bland-Altman plot in Fig. 2. The LOA was wide (± 1797 kJ) and the plot showed a bias towards overestimation at higher means (y = 0.31x – 716; *r* = 0.34, *P* = 0.038).

**Fig. 3:**
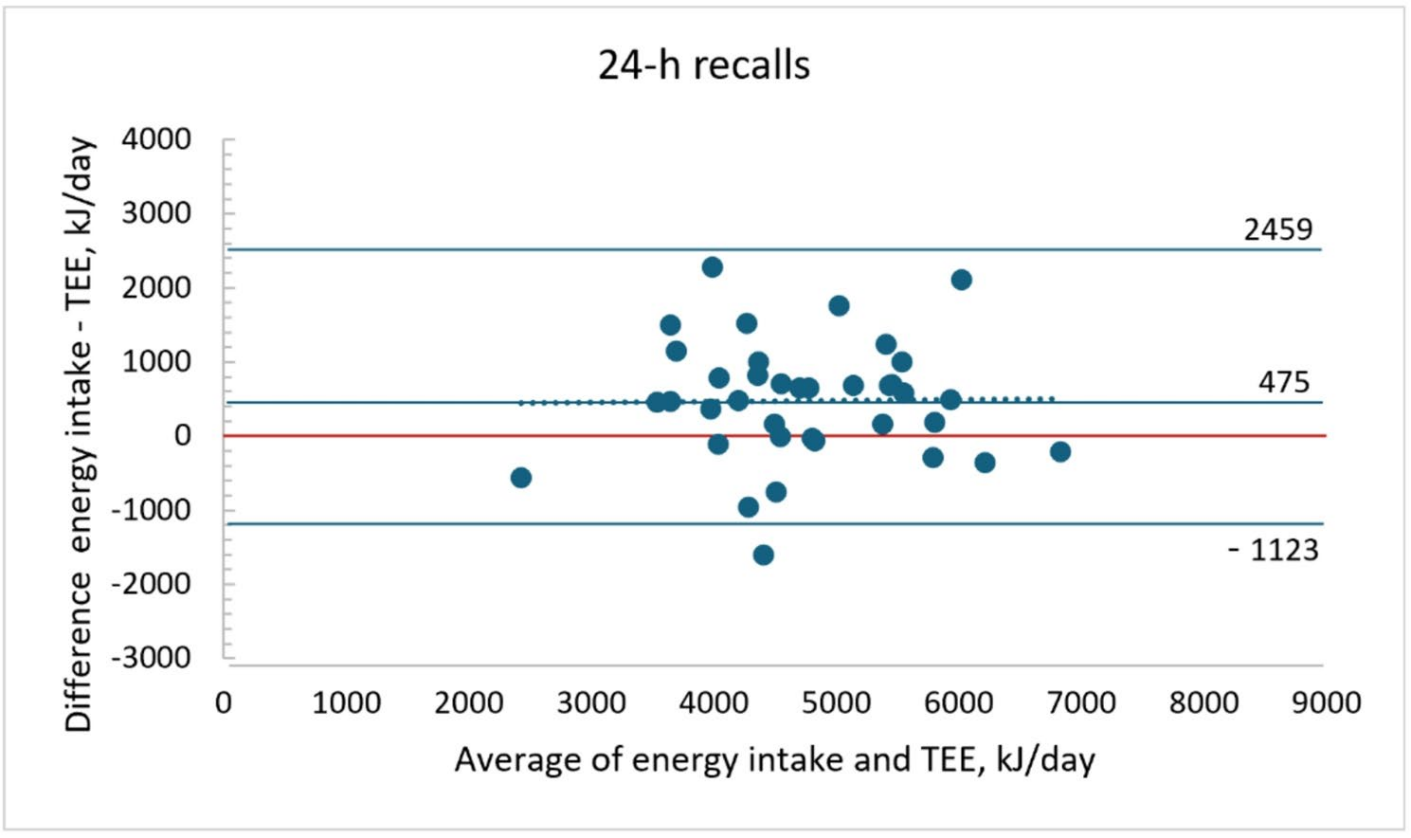
Bland-Altman plot of reported energy intake with the 24-h dietary recalls (mean of two days) and total energy expenditure by doubly labelled water (TEE) (*n* = 37)

For comparison the reported energy intake from the 24-h dietary recalls was also evaluated against TEE. Energy intake was 5084 (1006) kJ and also over reported (475 kJ, 15%, *P* = 0.001). The correlation between reported energy intake and TEE was 0.66. The LOA in the Bland-Altman plot were also wide ± 1598 kJ (Fig. 3), but no systematic bias at higher intake levels was observed (y = 0.01x + 423; *r* = 0.03, *P* = 0.839).

The DLW subsample was also stratified by younger (< 37 months, *n* = 17) and older (≥ 37 months, *n* = 20) children. The Bland-Altman plots, displayed in Supplementary Material 3, were similar to the plots in the combined group. However, RiksmatenFlex overestimated energy intake most in the older age group.

### Relative validity of reported dietary intake by RiksmatenFlex

Intake per day of energy, macronutrients, added sugars, dietary fibre, wholegrains and the nutrients vitamin C, vitamin D, iron and calcium assessed using RiksmatenFlex and the reference method 24-h dietary recalls are displayed in Table 2. Reported energy intake was significantly higher with RiksmatenFlex than with the 24-h dietary recalls (median difference 194 kJ; IQR 708 kJ; *P* < 0.001) and the intake from protein, fat, and carbohydrates were all significantly higher (*P* = 0.012; *P* = 0.002 and *P* = 0.037, respectively). The proportions of energy from the macronutrients were however similar between the methods, as well as absolute and energy-standardised intake of added sugars, dietary fibres, wholegrains, vitamin C and vitamin D. On the other hand, calcium intake, both absolute and standardised for energy intake, was higher and energy-standardised iron intake lower, by Riksmaten Flex than with the 24-h dietary recalls. The spearman rank correlations between the two methods were strong, ranging from 0.73 (intake of wholegrains) to 0.90 (energy-standardised dietary fibre).

**Table 2:** Reported intake per day of energy and selected nutrients assed by RiksmatenFlex^a^ and 24-h dietary recalls^b^ in all children (*n* = 94)

	RiksmatenFlex	24-h recalls		
	Median (IQR)	Median (IQR)	*P* ^c^	*r*_s_^d^
Energy, kJ	5154 (2077)	4995 (1661)	0.001	0.87
Protein, g	44.0 (17.8)	40.9 (14.0)	0.012	0.85
Protein E%^e^	14.3 (2.8)	13.9 (3.2)	0.845	0.77
Fat, g	45.7 (22.1)	44.8 (19.0)	0.002	0.87
Fat, E%^5^	33.7 (8.2)	33.0 (5.9)	0.070	0.79
Carbohydrates, g	144 (56)	140 (52)	0.037	0.87
Carbohydrate, E%^e^	49.7 (7.8)	50.0 (6.7)	0.080	0.80
Added sugars, g	16.8 (17.5)	17.0 (19.8)	0.931	0.89
Added sugars, E%^e^	5.6 (5.4)	6.0 (6.5)	0.359	0.87
Dietary fibre, g	14.4 (6.9)	13.6 (6.0)	0.082	0.91
Dietary fibre, g/1000 kJ	2.8 (1.2)	2.8 (1.2)	0.385	0.90
Wholegrains, g	22 (23)	24 (22)	0.869	0.73
Vitamin C, mg	61 (55)	67 (48)	0.528	0.83
Vitamin D, µg	7.3 (6.9)	6.7 (7.1)	0.487	0.89
Iron, mg	5.4 (2.5)	5.7 (2.6)	0.291	0.89
Calcium, mg	584 (385)	564 (252)	< 0.001	0.89

^a^ Food diaries, mean of two days

^b^ mean of two days

^c^Wilcoxon signed rank test

^d^Spearman rank correlations, all *P* < 0.001

^e^Percent of total energy intake

The agreement between the two dietary methods was also substantial for most of the investigated nutrients (weighted kappa, K_w_ ranging between 0.61 and 0.80). The exception was proportion of energy from protein and fat as well as wholegrain intake where the agreement was moderate (K_w_ 0.41–0.60) and vitamin D where the agreement was almost perfect (K_w_=0.81). Details of the agreement analyses are presented in Supplementary Material 2 (Table S2).

The reported intake of selected food groups with the two dietary methods are displayed in Table 3. The combined food group fruit and vegetables was higher with RiksmatenFlex than with the 24-h dietary recalls (247 g versus 227 g; *P* = 0.029). Processed meat intake was also higher with RiksmatenFlex than with the 24-h dietary recalls, although the mean difference was very small (20 g versus 19 g; *P* = 0.007). None of the other included food groups differed between the dietary assessment methods. The Spearman rank correlations between the two methods were strong and ranged from 0.81 for vegetables to 0.98 for baby foods. The agreement was also almost perfect (Kw 0.81-1.00) for milk and cheese products, fruit juice, beverages, candy and chocolates, desserts and baby foods whereas the agreement was substantial (Kw 0.61–0.80) for the other investigated food groups. See Supplementary Material 2 (Table S3) for more details.

**Table 3:** Median intakes (gram), interquartile range (IQR) and correlations for food groups estimated by RiksmatenFlex^a^ and 24-h dietary recalls^b^ for all children (*n* = 94)

Food group	RiksmatenFlex	24-h dietary recalls		
	Median (IQR)	Median (IQR)	*P* ^c^	*r*_s_^d^
Fruits^e^	167 (152)	160 (146)	0.060	0.90
Vegetables^e^	68 (81)	70 (70)	0.192	0.81
Fruits and vegetables^e^	247 (169)	227 (152)	0.029	0.88
Red meat^f^	8.5 (21)	8.1 (25)	0.676	0.84
Processed meat^f^	19 (57)	20 (50)	0.007	0.93
Milk and yoghurts	129 (227)	129 (218)	0.648	0.92
Cheese incl. dishes	7.5 (21)	5.0 (20)	0.138	0.92
Bread	46 (38)	46 (46)	0.583	0.87
Fruit juice	0 (0)	0 (0)	0.287	0.84
Beverages	0 (75)	0 (75)	0.214	0.93
Candy and chocolates	0 (6.5)	0 (5.5)	0.514	0.90
Sweet pastries	3.6 (15)	2.5 (15)	0.848	0.87
Desserts	0 (15)	0 (13)	0.848	0.85
Baby foods	0 (120)	0 (125)	0.308	0.98

^a^Food diaries, mean of two days

^b^mean of two days

^c^Wilcoxon signed rank test

^d^Spearman rank correlation

^e^including fruits/vegetables from composite dishes

^f^including meat from composite dishes

### Dietary intake differences by age group

As the age range in the study was large, the analyses were also stratified by less than 37 months (*n* = 44) and 37 months and older (*n* = 50) in an exploratory analysis. Similar differences in dietary intake as in the total sample were observed between the two dietary methods in the older age group, whereas differences in reported dietary intake between the two methods were small in the younger age group (Supplementary Material 2, Tables S4–S7).

### Participants’ perception of the two dietary assessment methods

Of the 91participants who answered the question on preferred dietary method, 74 participants preferred RiksmatenFlex. Eight preferred the 24-h dietary recall interview, seven preferred a combination of the two, and two participants did not have an opinion either way. Most parents found both methods easy to report food intake in. The average (min-max) score was 2.6 (1-8) for RiksmatenFlex and 3.1 (1-10) for the 24-h dietary recalls (on a scale from 1 -very easy to 10 - very difficult). Parents spent on average (min-max) 18 (5–80) minutes per day for the reporting of food intake in RiksmatenFlex whereas the time spent on the 24-h dietary recall interview was on average 24 (15–50) minutes.

The participants’ parents reported that RiksmatenFlex was easy to use in terms of adding meals, searching for foods and assessing portion sizes from the pictures provided. Some parents would have preferred to add the amounts in weight instead of household measurements or from portion pictures. About a third of the participants (*n* = 27) said that they couldn’t find a specific food in the food list on RiksmatenFlex. Depending on the food, they added something similar instead, did not add the food at all, or added the ingredients instead of a composite dish. A few requested a text box where they could add foods that they couldn’t find.

Many parents found it easier to report foods in the food diary than in the interview since it was not dependent on memory. The fact that the food diary could be filled in during the day when it suited the parents was also appreciated. Many reported that it was difficult to remember all foods during the interviews but some reported that the interview captured more details since there were more follow-up questions.

## Discussion

This is the first validation study of the web-based dietary assessment method RiksmatenFlex in young children. DLW measured TEE and 24-h dietary recalls were used as reference methods. The results showed that energy intake was overestimated with RiksmatenFlex compared to TEE. Energy intake was higher with RikmatenFlex than with the 24-h dietary recalls, but dietary intakes were, with some exceptions, similar with the two dietary assessment methods. Parents found it easy to report food intake in RiksmatenFlex, and a majority preferred the web-based application over interviews.

### Validation of energy intake

In the earlier validation studies of RiksmatenFlex, energy intake was overestimated in adolescents compared to estimated TEE [12], but not in pregnant women compared to DLW measured TEE [13]. However, in the earlier studies energy intake was recorded retrospectively whereas in the present study RiksmatenFlex was used as a food diary and food intake was recorded prospectively. Food diary methods tend to produce underestimation of energy intake compared to DLW measured TEE in children [1]. Although, the underestimation of energy intake by food diaries seems to be smaller in young children (≤ 6 years) [1].

The LOA from RiksmatenFlex were wide, indicated poor agreement at the individual level. Wide LOA were also observed by the 24-h dietary recalls and this is also reported from most validation studies using DLW as a reference method, in both adults [4] and children [8]. Food records and dietary recall methods generally include only a few days and some discrepancy at the individual level would be expected as a few days are not representative of habitual energy intake whereas the DLW measurement covers 1–2 weeks. To reduce the effect of the day-to-day variation in the current study, reported energy intake was transformed into usual intake using the MSM [18, 19]. This was considered appropriate to do as transformations into habitual intake is commonly done when presenting results from dietary surveys [22, 23]. The LOA were, however, still wide using MSM-adjusted energy intake data but similar [21, 24–26], or narrower [27–29] compared to previously reported LOA in validation studies of recording and recall methods compared with the DLW method. The mean difference between observed energy intake by RiksmatenFlex and TEE also became larger at higher energy intake levels, but this bias was not observed for the 24-h dietary recalls. This bias is commonly reported from validation studies of energy intake in children [12, 20, 24, 27, 30, 31], but not all [21, 26].

### Relative validity of dietary intake

Overall RiksmatenFlex provided dietary intake estimates that were comparable with the estimates from the 24-h dietary recalls. However, there are some exceptions to note. In line with the higher energy intake reported by RiksmatenFlex, absolute intakes of protein, fat and carbohydrates were all higher by RiksmatenFlex than by the 24-h dietary recalls. Furthermore, intakes of calcium and the combined food group fruit and vegetables were also higher by RiksmatenFlex than by the 24-h recalls. The difference in energy intake was larger in the older children (≥ 37 months) than in the younger children (< 37 months) and this is also in line with the results from the DLW sub study where overestimation of energy intake was particularly seen in the older age group. Shared responsibility between parents and preschool teachers may compromise the dietary reporting accuracy [32] and it may be speculated that this effects the reporting bias more with RiksmatenFlex than with the interviews in the older age group. The correlations between food groups were similar to those reported in the validation study of the photo-recording method TECH [21] or stronger than those previously reported in validation studies of young children [20, 31]. The agreement between the methods was also supported by weighted kappa statistics showing substantial or almost perfect agreement for most nutrients and food groups. The good agreement between the dietary methods was seen despite the large differences in detail between the two methods. The number of foods in RiksmatenFlex is limited and portion-sizes are predefined. In contrast, there are many more foods to choose from in 24-h dietary recall interviews that can be described in more detail through follow-up questions. On the other hand, the 24-h dietary recalls covered the same days as RiksmatenFlex and good agreement would be expected. Still, it is important to stress that reporting biases are likely to be correlated and that good agreement between the two methods does not necessarily reflect valid dietary intake [33]. Nevertheless, the results suggest that RiksmatenFlex’s ability to rank individuals by dietary intake is similar to the 24-h dietary recall method’s ranking ability.

### Methodological considerations

A major strength of this study is that energy expenditure was objectively measured using the DLW method [9, 34]. The high costs of this method limited the number of children that could be included in the subsample. Although the number of children included in the subsample was similar to most recent DLW studies in children [1, 8], there was not enough statistical power for subgroup analyses by both age and sex. Another limitation is the comparisons of dietary intake between the two dietary assessment methods. Both dietary assessment methods covered the same days, but as the recall interview covered the same day as the recorded day it was not possible to administer the dietary assessment methods in a random order. Thus, the parents had focused on their child’s diet before they were interviewed and this could have influenced the interview responses. Still, portion size estimations could still be tested between the two methods. The study is also limited by using a convenience sample not representative of parents in the general population. The participating parents had a higher educational background compared to parents in the general population [2] and it is likely that taking part in this study reflects an interest in nutrition. Most primary contact parents were also the children’s mothers where a majority had a higher education level. Thus, it is likely that the obtained dietary patterns and the attention to details are better than what would be expected from parents with a lower socioeconomic background and less interest in nutrition. Unfortunately, many studies aiming at the general population are also biased towards participants with a higher educational attainment. For example, in the most recent national dietary survey of young children in Sweden, Riksmaten Young Children, participating parents had a much higher educational attainment than the general Swedish population [2]. The results of this validation study may therefore be applicable to the Riksmaten Young Children and similar studies sampling participants from the general population, although further studies of the validity of RiksmatenFlex in parents of lower educational attainments are warranted.

## Conclusion

Previous validation studies have confirmed RiksmatenFlex as a reliable tool for collecting dietary intake data in intervention studies and national dietary surveys among adolescents and pregnant women. In this study, RiksmatenFlex demonstrated reasonable validity in young children and was well accepted by parents. These findings support its use in large-scale studies of young children, although potential overreporting of energy intake should be considered when interpreting results.

## Supplementary Information

Supplementary Material 1.

Supplementary Material 2.

Supplementary Material 3.

## Data Availability

The datasets used and analysed during the current study are available from the corresponding author on reasonable request.

## Abbreviations

DLW: Doubly labelled water
EFSA: European Food Safety Authority
E%: Percent of total energy intake
MSM: Multiple Source Method
SFA: Swedish Food Agency
TEE: Total Energy Expenditure
