# Supplementary material for: Validation of the web-based dietary assessment tool (RiksmatenFlex) against doubly labelled water and 24-h dietary recalls in Swedish pre-school children
Source: Nutr J. 2026 Mar 17;25:40. doi: 10.1186/s12937-026-01315-9 (PMC13063513; doi:10.1186/s12937-026-01315-9)
Supplement: Supplementary file 1 — Supplementary Material 1. [file 12937_2026_1315_MOESM1_ESM.pdf]

Examples from RiksmatenFlex

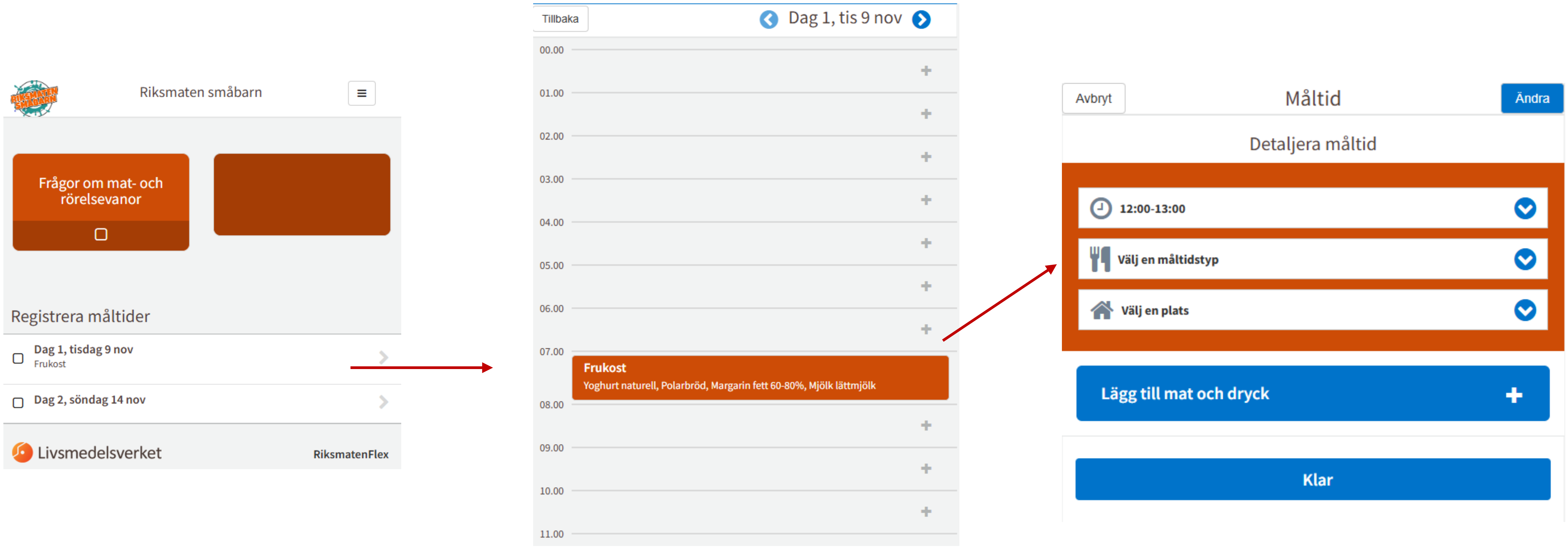

Supplementary Figure 2a. Web page, specification of meal type, time, place and food items.

Examples from RiksmatenFlex

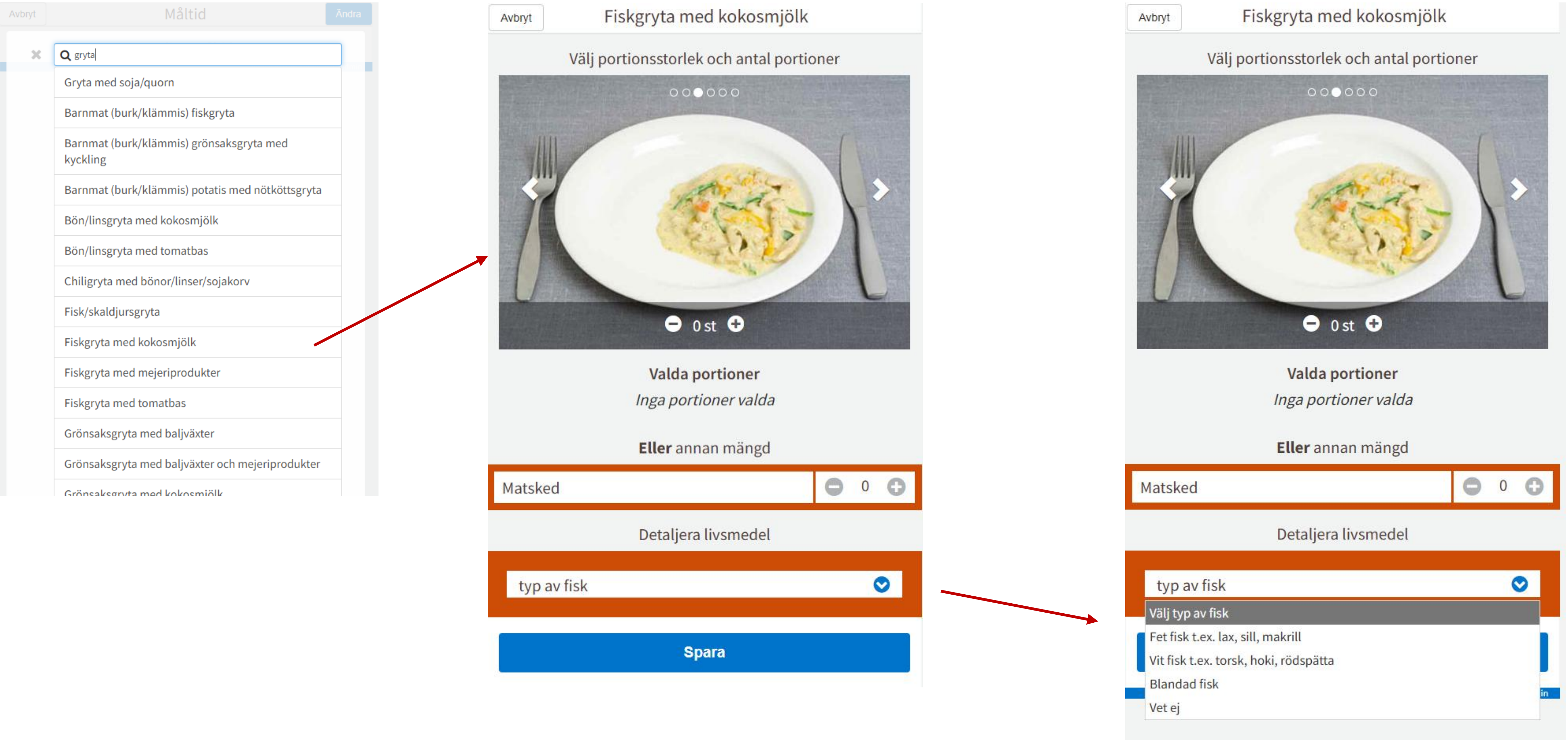

Supplementary Figure 2b. Specification of portion sizes and where appropriate type of fish or meat in mixed dishes.

Examples from RiksmatenFlex

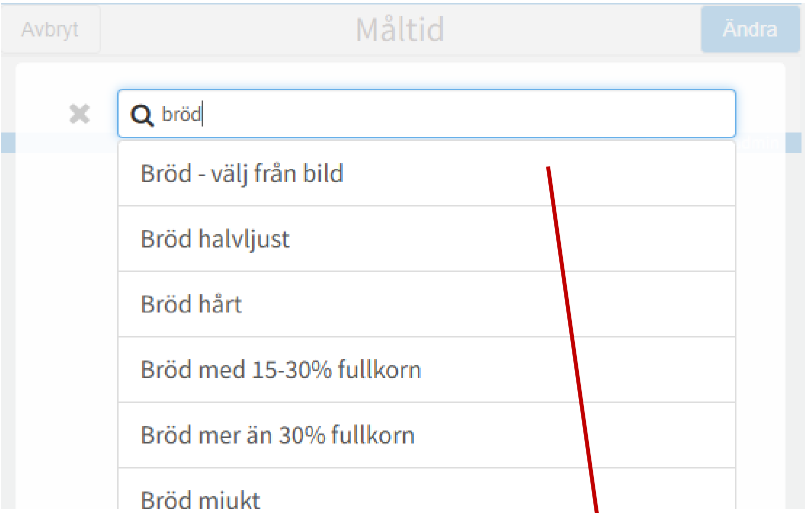

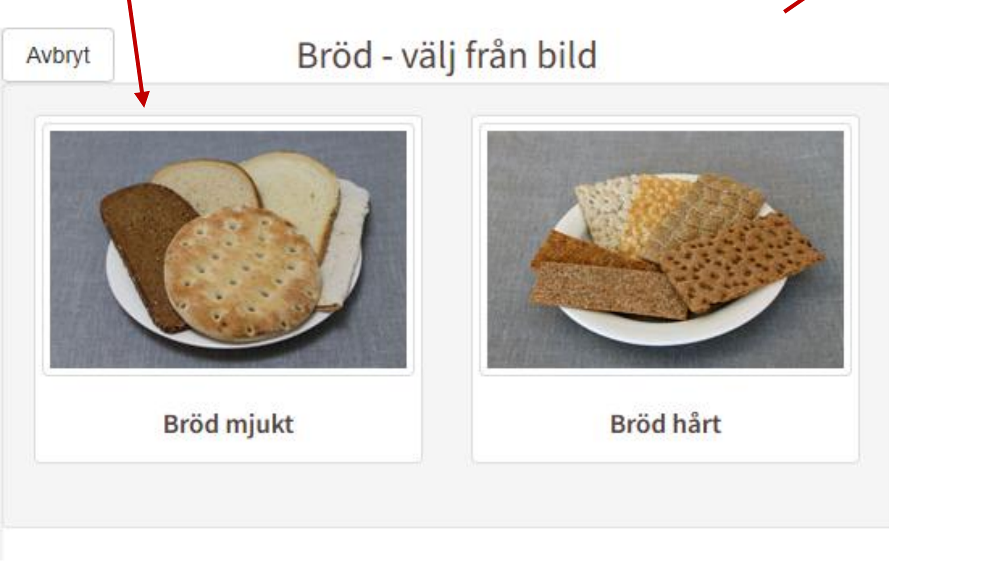

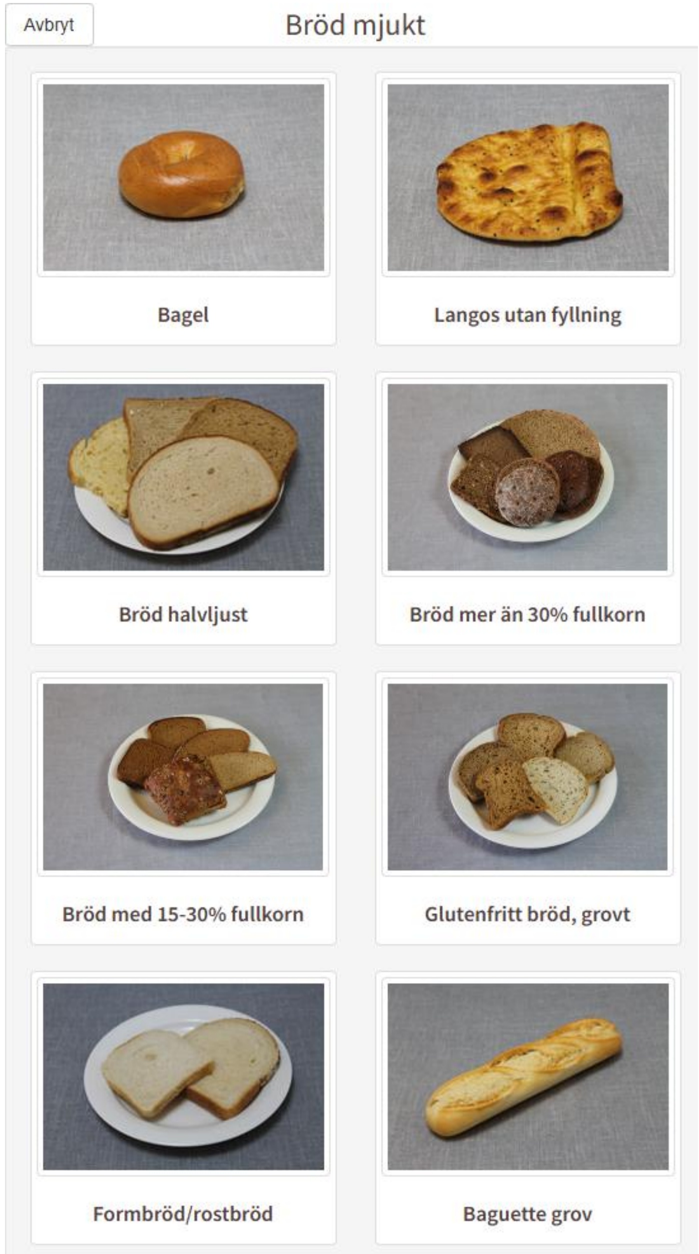

Supplementary Figure 2c. Specification of bread types.
