# Supplementary material for: Validation of the web-based dietary assessment tool (RiksmatenFlex) against doubly labelled water and 24-h dietary recalls in Swedish pre-school children
Source: Nutr J. 2026 Mar 17;25:40. doi: 10.1186/s12937-026-01315-9 (PMC13063513; doi:10.1186/s12937-026-01315-9)
Supplement: Supplementary file 2 — Supplementary Material 2. [file 12937_2026_1315_MOESM2_ESM.pdf]

Supplementary tables

Table S1. Description of food groups.

**Food group**	**Includes**
Fruits	Fruits and berries eaten as such and the amounts from composite dishes like smoothie and apple pie
Vegetables	Vegetables and root vegetables eaten as such and the amounts from composite dishes like curry stew and vegetable patties
Fruits and vegetables	The total amount of fruits and vegetables
Red meat	All meat from pork, beef, lamb and game including the amounts from composite dishes like meat stew, pulled pork and bolognese
Processed meat	All processed meat products including the amounts from composite dishes e.g. sausage, ham and paté
Milk and yoghurts	All kinds of yoghurt and fermented products like sour milk
Cheese incl. dishes	All kinds of cheese and dishes like fried halloumi and halloumi stew
Bread	All kinds of crisp bread, soft bread and savoury biscuits
Fruit juice	All kinds of fruit juices
Beverages	All kinds of sweet beverages including hot chocolate and beverages with artificial sweeteners
Candy and chocolates	All kinds of sweets, candy and chocolate confectionary
Sweet pastries	Sweet biscuits, cakes and sponge layer cakes
Desserts	Desserts, ice cream, sweet soups and sauces
Baby foods	Porridge, gruel, infant formula and preserved baby foods

Table S2. Percentages of children classified into the same and opposite thirds of energy and nutrient intake, and weighted kappa (Kw) by Riksmaten Flex and 24-h recalls in 94 children.

	Percentage classified in				
	**Same third [a]**	**Opposite third [b]**	**K_w**	**95% CI[c]**	**Agreement[d]**
Energy, kJ	73	1	0.69	0.56 - 0.79	Substantial
Protein, g	74	2	0.69	0.56 - 0.79	Substantial
Protein E%[d]	64	2	0.57	0.44 - 0.69	Moderate
Fat, g	72	0	0.69	0.57 - 0.78	Substantial
Fat, E%[d]	57	0	0.50	0.38 - 0.63	Moderate
Carbohydrates, g	77	0	0.74	0.61 - 0.83	Substantial
Carbohydrate, E%[d]	66	2	0.59	0.47 - 0.73	Moderate
Added sugars, g	74	0	0.71	0.61 - 0.81	Substantial
Added sugars, E%[d]	68	0	0.64	0.52 - 0.75	Substantial
Dietary fibre, g	74	0	0.71	0.60 - 0.81	Substantial
Dietary fibre, g/1000 kJ	77	0	0.74	0.63 - 0.84	Substantial
Wholegrains, g	65	7	0.50	0.35 - 0.64	Moderate
Vitamin C, mg	71	1	0.67	0.54 - 0.78	Substantial
Vitamin D, µg	83	0	0.81	0.72 - 0.89	Almost perfect
Iron, mg	71	1	0.67	0.55 - 0.77	Substantial
Calcium, mg	72	0	0.69	0.57 - 0.79	Substantial

[a] Percentage of children classified in the same tertile
[b] Percent of children classified in the opposite tertile
[c] Strength of agreement: 0.00–0.20 (slight), 0.21–0.40 (fair), 0.41–0.60 (moderate), 0.61–0.80 (substantial), 0.81–1.00 (almost perfect).
[d] 95% CI, 95% confidence intervals
[e] Percent of total energy intake

Table S3. Percentages of children classified into the same and opposite thirds of food group intake, and weighted kappa (Kw) by Riksmaten Flex and 24-h recalls in 94 children.

	**Percentage classified in**				
	**Same third [a]**	**Opposite third [b]**	**K_w**	**95% CI[c]**	**Agreement[d]**
Fruit	79	2	0.74	0.62 - 0.84	Substantial
Vegetables	73	3	0.67	0.55 - 0.79	Substantial
Fruits and vegetables, g/d	76	1	0.71	0.60 - 0.83	Substantial
Red meat, g/d	79	2	0.74	0.63 - 0.84	Substantial
Processed meat, g/d	81	0	0.79	0.69 - 0.88	Substantial
Milk and yoghurts, g/d	86	2	0.82	0.71 - 0.91	Almost perfect
Cheese incl. dishes	89	1	0.87	0.79 - 0.94	Almost perfect
Bread	78	0	0.76	0.66 - 0.85	Substantial
Fruit juice	94	6	0.81	0.66 - 0.96	Almost perfect
Beverages	87	2	0.84	0.74 - 0.92	Almost perfect
Candy, chocolates	90	3	0.86	0.76 - 0.95	Almost perfect
Sweet pastries	84	3	0.80	0.68 - 0.89	Substantial
Desserts	93	7	0.82	0.70 - 0.95	Almost perfect
Baby foods	98	1	0.97	0.89 - 1.00	Almost perfect

[a] Percentage of children classified in the same tertile
[b] Percent of children classified in the opposite tertile
[c] Strength of agreement: 0.00–0.20 (slight), 0.21–0.40 (fair), 0.41–0.60 (moderate), 0.61–0.80 (substantial), 0.81–1.00 (almost perfect).
[d] 95% CI, 95% confidence intervals

Table S4. Median and interquartile range (IQR) of reported intake per day of energy and selected nutrients assed by RiksmatenFlex and 24-h dietary recalls in children younger than 37 months (n=44).

	**RiksmatenFlex**	**24-h dietary recalls**		
	**Median (IQR)**	**Median (IQR)**	**P[a]**	**r_s[b]**
Energy, kJ	4622 (1567)	4488 (1722)	0.203	0.88
Protein, g	35.8 (17.2)	37.5 (16.8)	0.273	0.85
Protein E%[c]	14.2 (3.1)	13.6 (2.9)	0.849	0.76
Fat, g	38.7 (21.9)	38.6 (18.9)	0.208	0.86
Fat, E%[c]	33.6 (8.0)	33.8 (5.4)	0.733	0.73
Carbohydrates, g	122 (43)	125 (43)	0.395	0.87
Carbohydrate, E%[c]	50.0 (8.0)	49.9 (5.5)	0.631	0.77
Added sugars, g	12.6 (11.5)	10.2 (11.8)	0.357	0.84
Added sugars, E%[c]	4.5 (4.1)	4.4 (4.6)	0.462	0.82
Dietary fibre, g	12.4 (5.6)	11.6 (4.8)	0.795	0.87
Dietary fibre, g/1000 kJ	2.9 (1.0)	2.9 (1.1)	0.491	0.89
Wholegrains, g	22 (22)	24 (22)	0.949	0.80
Wholegrains, g/1000 kJ	4.8 (5.1)	5.6 (4.2)	0.922	0.72
Vitamin C, mg	60 (46)	74 (49)	0.484	0.83
Vitamin C, g/1000 kJ	15 (9)	15 (10)	0.212	0.82
Vitamin D, µg	9.5 (10.4)	8.5 (11.2)	0.682	0.92
Vitamin D, g/1000 kJ	1.9 (2.8)	2.0 (2.9)	0.278	0.94
Iron, mg	5.6 (2.8)	6.0 (3.3)	0.363	0.92
Iron, mg/1000 kJ	1.4 (0.8)	1.0 (0.4)	0.014	0.95
Calcium, mg	546 (229)	513 (231)	0.258	0.84
Calcium, mg/1000 kJ	125 (50)	120 (54)	0.528	0.77

[a] Wilcoxon signed rank test; [b] Spearman correlation; [c] Percent of total energy intake.

Table S5. Median and interquartile range (IQR) of reported intake per day of energy and selected nutrients assed by RiksmatenFlex and 24-h dietary recalls in children 37 months and older (n=50).

	**RiksmatenFlex**	**24-h dietary recalls**		
	**Median (IQR)**	**Median (IQR)**	**P[a]**	**r_s[b]**
Energy, kJ	6032 (1623)	5492 (1387)	0.002	0.79
Protein, g	49.2 (17.2)	46.7 (12.3)	0.019	0.78
Protein E%[c]	14.3 (3.4)	14.1 (3.9)	0.909	0.78
Fat, g	51.6 (23.1)	49.3 (15.5)	0.002	0.83
Fat, E%[c]	34.0 (9.0)	32.5 (6.5)	0.027	0.84
Carbohydrates, g	160 (63)	156 (57)	0.040	0.80
Carbohydrate, E%[c]	50.0 (8.3)	50.2 (7.3)	0.054	0.81
Added sugars, g	21.8 (22.8)	22.4 (22.1)	0.534	0.85
Added sugars, E%[c]	6.9 (7.2)	8.0 (6.6)	0.058	0.86
Dietary fibre, g	15.1 (7.0)	15.2 (7.3)	0.040	0.90
Dietary fibre, g/1000 kJ	2.7 (1.2)	2.8 (1.2)	0.681	0.90
Wholegrains, g	21 (26)	26 (23)	0.826	0.70
Wholegrains, g/1000 kJ	3.2 (4.3)	4.4 (4.8)	0.291	0.68
Vitamin C, mg	61 (57)	57 (43)	0.856	0.83
Vitamin C, g/1000 kJ	10 (9)	11 (9)	0.152	0.80
Vitamin D, µg	6.6 (4.8)	6.0 (4.1)	0.175	0.80
Vitamin D, g/1000 kJ	1.0 (0.8)	1.1 (0.7)	0.924	0.77
Iron, mg	5.3 (2.3)	5.5 (2.0)	0.605	0.87
Iron, mg/1000 kJ	0.9 (0.4)	0.9 (0.4)	0.009	0.89
Calcium, mg	668 (406)	624 (370)	<0.001	0.92
Calcium, mg/1000 kJ	126 (71)	119 (54)	0.009	0.91

[a] Wilcoxon signed rank test; [b] Spearman correlation; [c] Percent of total energy intake.

Table S6. Median intakes (gram), interquartile range (IQR) and correlations for food groups assessed by RiksmatenFlex and 24-h dietary recalls in children <37 months (n=44).

	**RiksmatenFlex**	**24-h dietary recalls**		
**Food group**	**Median (IQR)**	**Median (IQR)**	**P[a]**	**r_s[b]**
Fruits[c]	170 (154)	163 (150)	0.411	0.94
Vegetables[c]	59 (66)	72 (54)	0.506	0.79
Fruits and vegetables[c]	234 (148)	224 (128)	0.333	0.90
Red meat[c]	5.5 (20)	6.5 (24)	0.962	0.85
Processed meat[c]	7.2 (39)	11.9 (30)	0.184	0.92
Milk and yoghurts	88 (171)	88 (208)	0.969	0.94
Cheese incl. dishes	5.5 (26)	5.0 (28)	0.915	0.94
Bread	39 (25)	38 (27)	0.617	0.78
Fruit juice	0 (0)	0 (0)	0.031	0.87
Beverages	0 (41)	0 (25)	0.883	0.81
Candy and chocolates	0 (2.2)	0 (2.2)	0.514	0.89
Sweet pastries	0 (10)	0 (5.8)	0.254	0.85
Desserts	0 (0)	0 (0)	0.941	0.64
Baby foods	118 (314)	121 (293)	0.719	0.96

[a] Wilcoxon signed rank test; [b] Spearman correlation; [c] including fruits/vegetables or meat from composite dishes.

Table S7. Median intakes (gram), interquartile range (IQR) and correlations for food groups eassessed by RiksmatenFlex and 24-h dietary recalls in children ≥37 months (n=50).

	**RiksmatenFlex**	**24-h dietary recalls**		
**Food group**	**Median (IQR)**	**Median (IQR)**	**P[a]**	**r_s[b]**
Fruits[c]	165 (146)	152 (134)	0.103	0.87
Vegetables[c]	75 (109)	69 (95)	0.230	0.82
Fruits and vegetables[c]	269 (155)	240 (132)	0.041	0.84
Red meat[c]	12 (26)	8.1 (25)	0.619	0.84
Processed meat[c]	29 (63)	20.2 (50)	0.017	0.90
Milk and yoghurts	189 (262)	129 (218)	0.583	0.89
Cheese incl. dishes	7.5 (18)	5.0 (12)	0.048	0.91
Bread	59 (48)	52 (59)	0.283	0.88
Fruit juice	0 (0)	0 (12)	0.857	0.82
Beverages	0 (125)	8.2 (100)	0.099	0.95
Candy and chocolates	0 (12)	0 (12)	0.990	0.90
Sweet pastries	6.2 (18)	10 (21)	0.756	0.85
Desserts	0 (18)	0 (18)	0.818	0.95
Baby foods	0 (0)	0 (0)	0.312	0.99

[a] Wilcoxon signed rank test; [b] Spearman correlation; [c] including fruits/vegetables or meat from composite dishes.
