# Supplementary material for: Validation of the web-based dietary assessment tool (RiksmatenFlex) against doubly labelled water and 24-h dietary recalls in Swedish pre-school children
Source: Nutr J. 2026 Mar 17;25:40. doi: 10.1186/s12937-026-01315-9 (PMC13063513; doi:10.1186/s12937-026-01315-9)
Supplement: Supplementary file 3 — Supplementary Material 3. [file 12937_2026_1315_MOESM3_ESM.pdf]

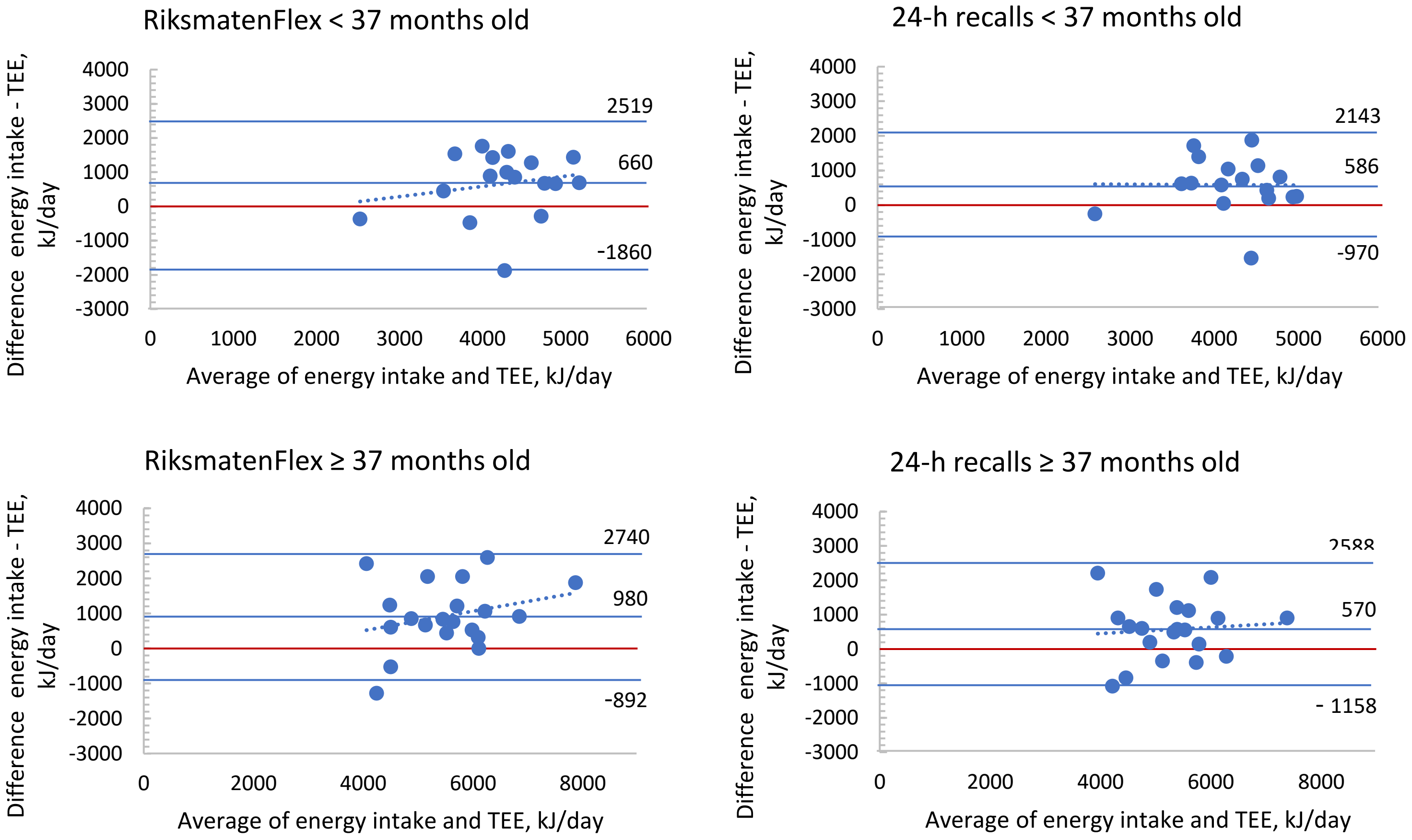

Supplementary Figure 1a – 1d. Bland-Altman plots of reported energy intake with RiksmatenFlex or 24-h dietary recalls and total energy expenditure by doubly labelled water (TEE) in children <37 months (n=17) and children ≥37 months (n=20).
